# Addition to “Bicyclopentylation of Alcohols with Thianthrenium Reagents”

**DOI:** 10.1021/jacs.4c00054

**Published:** 2024-01-11

**Authors:** Zibo Bai, Beatrice Lansbergen, Tobias Ritter

Page 25961. To our previous Communication, we would like to add ref 22c, which cites a thesis for a preliminary experiment for the alkylation of a 1,2-diol with reagent **1b**, albeit using a different catalyst and conditions.

The findings and conclusions of the original communication remain unchanged. We apologize for any inconvenience that this may have caused the readers of *JACS*.

(22) (c) Alvarez PariE. M. W.Reactivity of aryl sulfonium salts in Pd catalysis, cine-substitution besides redox-activation and disclosure of bicyclo[1.1.1]pentane sulfonium salts as bioisosteres of aryl sulfonium salts. PhD Thesis, RWTH Aachen University, Aachen, 2022.

